# Extraperitoneal laparoscopic resection for retroperitoneal lymphatic cysts: initial experience

**DOI:** 10.1186/s12894-017-0288-1

**Published:** 2017-11-13

**Authors:** Yichun Wang, Chen Chen, Chuanjie Zhang, Chao Qin, Ninghong Song

**Affiliations:** 1 The First Clinical Medical College, Nanjing Medical University, Nanjing, China; 2 Department of Urology, The First Affiliated Hospital of Nanjing Medical University, 300 Guangzhou Road, Nanjing, 210029 China

**Keywords:** Hydronephrosis, Lymphatic cyst, Laparoscope

## Abstract

**Background:**

To assess the safety and efficacy of laparoscopic retroperitoneal resection for retroperitoneal lymphatic cysts.

**Methods:**

A retrospective analysis was conducted based on clinical data from eight patients with hydronephrosis caused by retroperitoneal lymphatic cysts. All patients underwent laparoscopic retroperitoneal lymphatic cyst resection and received postoperative follow-up. A follow-up ultrasound was performed postoperatively every 6–12 months to evaluate the recovery of the hydronephrosis.

**Results:**

All operations were successful, and their postoperative pathological results revealed lymphatic cyst walls. The operation time ranged from 43 to 88 min (mean: 62 min), with a blood loss of 20 to 130 mL (mean: 76 mL), and the length of hospital stay was 3 to 6 days (mean: 4.5 days). Within the follow-up of 12 to 36 months (mean: 28.5 months), great relief was detected in all eight cases, and no recurrence was found. Moreover, complications such as renal pedicle or renal pelvis injury were not observed.

**Conclusions:**

Laparoscopic retroperitoneal lymphatic cyst resection is an effective treatment for retroperitoneal lymphatic cysts and has the advantages of being minimally invasive, producing less intraoperative blood loss and leading to a quick recovery. This treatment thus deserves further studies.

## Background

Lymphatic cysts are a rare lymphatic-vessel-generated disease that have a thick fibrotic wall lacking epithelial lining [1], and they generally occur following congenital lymphatic system heteroplasia or surgical procedures such as pelvic or retroperitoneal operations [2, 3]. There are no typical manifestations, and they are mostly diagnosed incidentally with physical examination or surgery [4]. Retroperitoneal lymphatic cysts are particularly uncommon and usually appear near the renal, retrocolon and cauda pancreatis [5, 6]. They do not cause any symptoms at first. When a cyst becomes sufficiently large, it could constrict the neighboring anatomic structures and cause symptoms such as lower abdominal pain, obstructive uropathy, lower lymphoedema, bowel obstruction and venous thrombosis [7–10]. Most patients come to the hospital for the presence of an abdominal mass. It is difficult to make a definite diagnosis before the operation. However, by using X-ray, computed tomography (CT), ultrasound, and other techniques, doctors could make a presumptive diagnosis. The narrow and deep retroperitoneal space increases the difficulty of the operation, so an open operation is always the first choice. However, with the development of laparoscopic techniques, laparoscopic retroperitoneal lymphatic cyst resection has become an optional choice and possesses advantages such as short hospitalization duration, less pain and short recovery time. It is a quite promising minimally invasive surgery [11]. From December 2011 to January 2014, 8 patients underwent laparoscopic retroperitoneal lymphatic cyst resection.

## Methods

### Clinical information

**Fig. 1:**
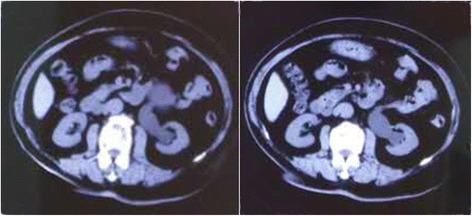
CT:Moderate hydronephrosis

**Fig. 2:**
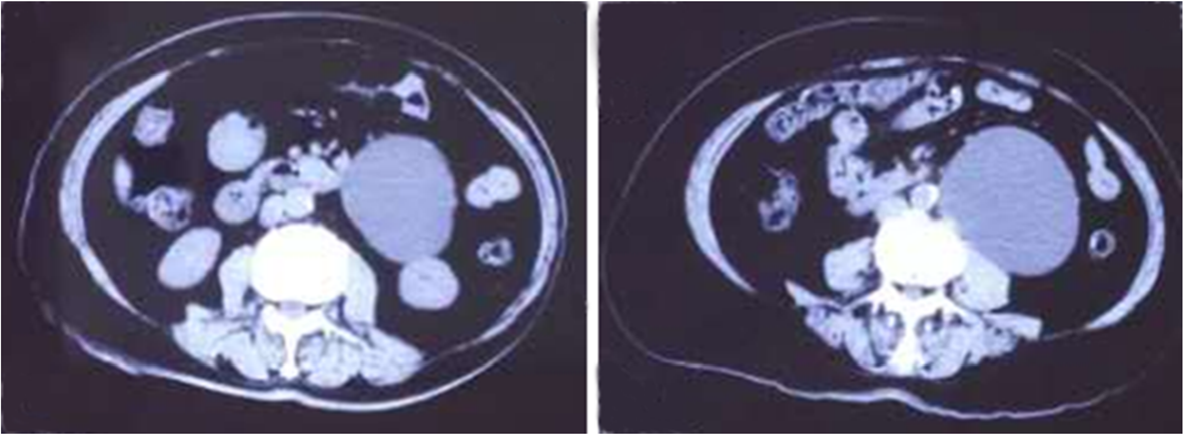
Retroperitoneal lymphatic cyst

From December 2011 to January 2014, 8 male patients with hydronephrosis caused by retroperitoneal lymphatic cysts were admitted to our hospital. Routine preoperative written informed consent was obtained from all patients involved in this study. The indication for laparoscopic retroperitoneal lymphatic cyst resection in this study was hydronephrosis accompanied by the obvious obstructive factor of a retroperitoneal lymphatic cyst. The patients’ ages ranged from 38 to 85 (mean of 57). Of all the cases, one patient had undergone an appendectomy in 2001, and the others declared no medical history of trauma or surgery. Two patients suffered waist discomfort. The diameter of the cyst ranged from 7.5 cm to 12.0 cm (mean of 9.7 cm). The degree of hydronephrosis was described according to ultrasound (Table 1). The preoperative serum creatinine levels were in the normal range. All patients underwent preoperative examination, including ultrasound, enhanced CT scan, and intravenous urography (IVU) combined with other laboratory examinations, and were diagnosed with retroperitoneal lymphatic cyst with hydronephrosis (Figs. 1 and 2). The follow-up was 12–36 months. During the follow-up, ultrasound examination was performed every 6 months to monitor the development of hydronephrosis in the first year. Thereafter, ultrasound was performed every 12 months.

**Table 1:** Preoperative demographic data and information about patients

Patient	Age	Cyst side	Cyst diameter (cm)	Hydronephrosis stage	Symptom
1	38	Right	7.5	Mild	No symptom
2	67	Right	9.3	Moderate	No symptom
3	46	Left	8.2	Mild	No symptom
4	42	Right	8.7	Moderate	No symptom
5	72	Left	10.6	Severe	Waist discomfort
6	56	Left	12.0	Moderate	No symptom
7	85	Right	11.6	Severe	No symptom
8	52	Right	9.7	Severe	Waist discomfort

### Operation procedure

All patients were given general anesthesia through the trachea; then, a unilateral ureteral stent was inserted to identify and preserve ureter function during the operation. After introducing the ureteral sent, the patient was placed in the unaffected lateral position and tilted up to the waist bridge. A 2.0-cm incision was made to the inferior of the 12th rib in the posterior axillary line. Various muscular layers were bluntly divided until the peritoneum could be accessed. Then, a homemade balloon inflated with 700 mL of gas was inserted to create a retroperitoneal space. Using a forefinger, 0.5-cm, 0.5-cm, and 1.0-cm incisions were made into the inferior of rib in the anterior axillary line, near the crista iliaca, and 2 cm superior of crista iliaca in the midaxillary line, respectively. A 10-mm trocar was placed, and a pneumoperitoneum was created with a pressure of 10 mmHg. Then, the retroperitoneal fat was dissociated along the musculi psoas major until the diaphragm was reached. Subsequently, the perirenal fascia was exposed and incised from the anterior and lower renal poles. The musculi psoas major was exposed to find the ureter along the interior of the musculi psoas major. The cyst behind the renal pelvis was carefully circumferentially dissected from the ureter, surrounding vessels and adhesions. A titanium clip was used when the surrounding adhesions were difficult to dissociate. After successful dissociation, a small incision was made into the surface of the cyst to decompress the cyst. The liquid content was clear without evidence of bile, blood or chyle. Then, the cyst wall was excised completely and sent for pathological examination. Bleeding was then checked, and a drainage tube was inserted in the upper section of the renal surrounding. The ureteral stent was unsheathed 1 month after discharge from the hospital. Follow-up was performed regularly.

## Results

**Fig. 3:**
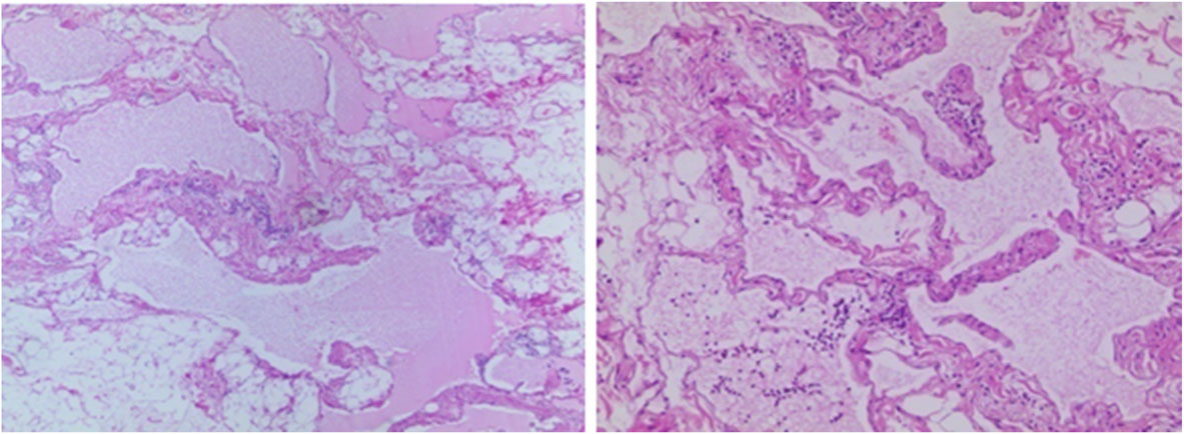
Postoperative pathology: lymphatic cyst, fibrous capsule wall

In these cases, all operations were completed successfully, with no injury of the renal hilus or collection system, no conversions to open surgery and no intraoperative blood transfusion. The operation time ranged from 43 to 88 min (mean: 62 min). The blood loss was 20–130 mL (mean: 76 mL). The perioperative hospitalization time was 3–6 days (mean: 4.5 days). The histopathologic results included lymphatic cyst with fibrous capsule walls (Fig. 3). The follow-up was 12–36 months (mean of 28.5 months). During the follow-up, no complications such as lymphatic fistula, renal pedicle or renal pelvis injury were observed. The hydronephrosis in all patients had resolved, and no recurrence was observed. The waist discomfort of two of the patients decreased (Table 2).

**Table 2:** Intraoperative and postoperative patient data

Patient	Age (year)	Hydronephrosis stage	Cyst diameter (cm)	Operative time (min)	Blood loss (mL)	Hospitalization time (days)	Follow-up (months)
1	38	Mild	7.5	43	20	3	18
2	67	Moderate	9.3	56	40	5	36
3	46	Mild	8.2	49	35	4	12
4	42	Moderate	8.7	62	79	5	24
5	72	Severe	10.6	88	130	6	36
6	56	Moderate	12.0	66	107	4	30
7	85	Severe	11.6	70	100	5	36
8	52	Severe	9.7	59	98	4	36

## Discussion

Retroperitoneal lymphatic cysts involve one or more chamber cysts with clear or chylous fluid. Most cysts are large but have no symptoms in the early stage; this is related to the anatomical characteristics of the retroperitoneal space, which is full of deep and large gaps. At the same time, the retroperitoneal lymphatic cyst grows slowly and shows no invasiveness. However, when the cyst grows too large, it could trigger some symptoms, such as infection, bleeding in the cyst, and constriction of the tissues, or even flatulence and hydronephrosis. Patients can occasionally palpate a painless mass on the abdomen [12]. The disease is easy to misdiagnose [13] and must be distinguished from other abdominal cysts, such as liver cysts, renal cysts, pancreas cysts, ovarian cysts, cystic teratoma and tumor cystic lesions [14]. Some studies revealed that retroperitoneal cysts could lead to the compression of the adjacent organs [15, 16]. Once a cyst is enlarged, it could compress the junction of the renal pelvis and ureter, which could result in obstruction, retention of urine and hydronephrosis. If the kidney does not contain a substantial lesion, the surgeon only needs to remove the cyst to relieve the hydronephrosis.

Several methods for the management of lymphatic cyst with various results have been proposed, including conservative observation, percutaneous catheter drainage with or without sclerotherapy and internal marsupialization. In the research of William E. Braun [17], the authors observed three cases of spontaneously drained lymphatic cysts over 1–2 weeks and adopted conservative observation treatment in three cases after renal transplantation. Their results hinted that there could be an alternative for managing asymptomatic or mildly symptomatic cases. This therapeutic schedule is based on the phenomenon that some surgically derived lymphatic cysts may cause minimal symptoms and spontaneously disappear over 1 year [17]. However, the background for this management is based mainly on surgically derived lymphatic cysts due to the destruction of lymphatic channels. Lymphatic vessels could regenerate over the time, and this process may explain the disappearance of some lymphatic cysts. In our research, most patients had no surgical or traumatic experience, and these cases are supposed to be classified as congenitally generated, which means that they may not be remediated without medical intervention [4]. Additionally, long-term obstruction of the ureter may lead to the deterioration of renal function, and should therefore be resolved in a timely manner. Among these therapies, many research institutions have adopted percutaneous catheter drainage because of its safety and efficacy. However, according to Jae-Kyu Kim [18], recurrence can be observed in 13% of patients after the first successful drainage procedure in the 6-month follow-up period. It has a long treatment duration: the mean duration of treatment ranges from 10 to 20 days, which increases patient inconvenience [1]. Additionally, during percutaneous catheter drainage, patients should undergo at least two lymphographic procedures, which have an associated radiation exposure. Internal marsupialization surgery also has limitations because it can only drain the sterile content into the peritoneal cavity and is not applicable for infected lymphatic cysts [19, 20]. The effect of retroperitoneal lymphatic cyst surgery is encouraging. During the surgery, the exact dissociation of all the adhesions around the cyst should be done carefully to ensure that all cyst walls are excised and thereby avoid recurrence. In our study, all patients had hydronephrosis that might have been caused by the obstruction, so we used minimally invasive surgery to remove the obstruction of the ureter. Laparoscopic surgery is safer, produces less pain after operation, produces less blood loss and leads to shorter hospitalization durations [11]. Laparoscopic retroperitoneal cystectomy can be done via two approaches: abdominal or retroperitoneal. This choice is based on the skill of the surgeon and the location of the cyst. Most retroperitoneal cysts grow near the dorsal side of the kidney, so the operation could be performed via the retroperitoneal approach, especially since the invention of the retroperitoneal balloon dilator, which provides surgeons with a clear view of the retroperitoneal structure and can create sufficient operation space. The retroperitoneal approach could avoid both injury to abdominal organs and abdominal contamination and could decrease the complications of bowel paralysis, adhesion and ejaculatory disorders [21]. The restrictions of laparoscopy because of a medical history of abdominal surgery, injury or infection could be overcome, and the damage to the pancreas and splenic vessels that can potentially occur when the pancreas is dissociated and turned over via a transperitoneal approach could also be avoided. In recent years, many domestic and foreign units have carried out retroperitoneal laparoscopic lymphatic cyst resection [11], and the effects of this operation are encouraging.

In our study, all patients underwent laparoscopic retroperitoneal lymphatic cyst resection via a retroperitoneal approach. All operations were successful. According to the treatment experience of our center, the main treatment regimen included the following steps: 1. The patients underwent a CT scan to determine the size of the cyst and the location of the renal pelvis and renal pedicle vessels. 2. During the operation, the surgeon paid attention to the retroperitoneal space, the liver, the duodenum, the colon, the pancreas, the spleen, the vena cava and other organs and vessels. To prevent bleeding, blunt dissection was performed. Where necessary, an ultrasonic knife was utilised to cut the adjacent tissue. 3. A drainage tube was placed in the case of hydronephrosis. 4. Intraoperative monitoring of blood oxygen saturation and carbon dioxide levels was used. This study is encouraging, but its application has some restrictions: The operation and equipment costs are high, we included only patients with hydronephrosis caused by retroperitoneal lymphatic cysts, and the number of the cases was limited. We need further research to validate this method.

## Conclusions

This study showed that laparoscopic retroperitoneal lymphatic cyst resection, with the advantages of being minimally invasive, producing less pain and having a short recovery time, may be an alternative method to cure hydronephrosis caused by retroperitoneal lymphatic cysts.

## Abbreviations

CT: Computed tomography
IVU: Intravenous urography
